# Acetabular Reconstruction in a Case of Chondrosarcoma

**DOI:** 10.7759/cureus.86433

**Published:** 2025-06-20

**Authors:** Daniel I Ríos Moreno, Francisco Mahaluf Recasens, Luis Bahamonde

**Affiliations:** 1 Medicine, Universidad San Sebastián, Concepción, CHL; 2 Hip Surgery, Complejo Asistencial Dr. Víctor Ríos Ruiz, Los Ángeles, CHL; 3 Orthopedics, Universidad de Chile, Santiago, CHL

**Keywords:** 3d printing in surgery, acetabular reconstruction, case report, custom implants, hip prosthesis, internal hemipelvectomy, orthopedic oncology, pelvic chondrosarcoma, skeletal reconstruction, surgical planning

## Abstract

Chondrosarcomas of the pelvis represent a challenge in clinical management because surgical resection is the only effective therapeutic option, given the resistance to radiotherapy and chemotherapy of malignant chondroid tumors. Reconstruction after resection involves critical structures, such as the acetabulum or hip joint, and is a demanding surgical procedure with high morbidity. Complications are frequent due to the complex anatomy of the pelvis, which on the one hand makes it difficult to obtain adequate surgical margins, and on the other hand, the various implants designed for reconstruction have shown a high rate of mechanical and infectious complications. In this report, we present an innovative technique for pelvic reconstruction after resection of the acetabular region (Enneking type II resection), whose planning included the use of a three-dimensional printed model (3D printing) of the patient's pelvis.

We describe a case of a 39-year-old patient with a grade 2 central chondrosarcoma. Surgical treatment included 3D model planning, followed by tumor resection and reconstruction of the acetabulum using conventional implants. The main mechanical support of the technique in this case was achieved using threaded Schanz wires as osteosynthetic fixation of the anterior and posterior columns, on which an acetabular basket was cemented. Although at six months, a fracture of the remaining iliac bone and slight elevation of the hemipelvis occurred, this did not mean pain or impairment of the ability to walk with a cane. This particular cost-effective technique allowed the functional reconstruction of the hip, at least in the medium term, so it can be considered as an alternative in resections in zone II of the pelvis. In comparison to customized 3D-printed implants, this approach represents a more cost-effective option, particularly in settings with limited resources. While personalized implants offer superior anatomic fit, they require specialized manufacturing and high costs, often limiting their accessibility. In contrast, this technique utilizes standard implants with planning aided by 3D models, allowing surgeons to tailor reconstructions without reliance on custom hardware. Given these advantages, the method may be adaptable to a broader range of complex pelvic resections where institutional resources are constrained.

## Introduction

Bone tumors in the pelvis constitute between 10% and 20% of primary malignant bone tumors [1,2]. Within the latter, chondrosarcoma is the third most common type, preceded only by multiple myeloma and osteosarcoma, accounting for one in five malignant bone tumors [3]. It mainly affects adults between 40 and 70 years of age, with a higher prevalence in men. The pelvis is the most common site, with the iliac bone being affected first, followed by the pubic bone and the ischium [1,4]. For tumors located in the acetabular region, corresponding to zone II of the Enneking classification, which includes the weight-bearing periacetabular portion of the pelvis, resection through an internal hemipelvectomy (a limb-sparing procedure that removes part of the pelvis without amputating the lower extremity) leaves a bone defect that is difficult to reconstruct if the objective is the restoration of a functional and stable hip [1,5].

The fabrication of 3D impressions of the particular clinical situation of each patient is a technique that has allowed better planning of the surgical resection, as well as the preoperative determination of the resulting bone defect. In this way, it is also possible to construct customized implants, adapted to the specific anatomy [6,7].

Recent literature has demonstrated that 3D printing technologies not only improve anatomical understanding and preoperative simulation but also significantly reduce intraoperative time, blood loss, and complication rates in pelvic and acetabular procedures, thereby contributing to better functional outcomes [8,9]. Although computer-aided design and computer-aided manufacturing (CAD-CAM)-fabricated implants may offer excellent anatomical fit, their cost and technical requirements limit their widespread application. In contrast, techniques like the one presented in this report, combining 3D planning with conventional implants, have been shown to be more accessible and economically feasible, particularly in resource-constrained settings [10].

This report describes a specific technique for pelvic reconstruction after en bloc resection of the acetabular region, corresponding to zone II of the Enneking classification. The procedure, known as internal hemipelvectomy, involves resection of one or more pelvic regions without amputation of the limb. In this case, surgical planning was carried out using a 3D-printed anatomical model, allowing reconstruction with conventional implants adapted to the patient’s anatomy. This report was aimed at highlighting a practical and cost-effective reconstructive approach for managing acetabular chondrosarcoma in settings without access to CAD-CAM prostheses.

## Case presentation

A 39-year-old male patient with no relevant morbid history presented to the emergency department with a six-month history of progressive left hip pain (coxalgia). The pain was initially intermittent but had become constant in recent weeks, limiting his mobility and causing nocturnal discomfort. During the last week, symptoms worsened significantly, prompting medical consultation. The patient also reported occasional difficulty with weight-bearing and a subjective sensation of instability. On physical examination, there was functional impotence of the left hip, with pain on passive mobilization and restricted range of motion. No overt swelling or leg length discrepancy was observed.

**Figure 1:**
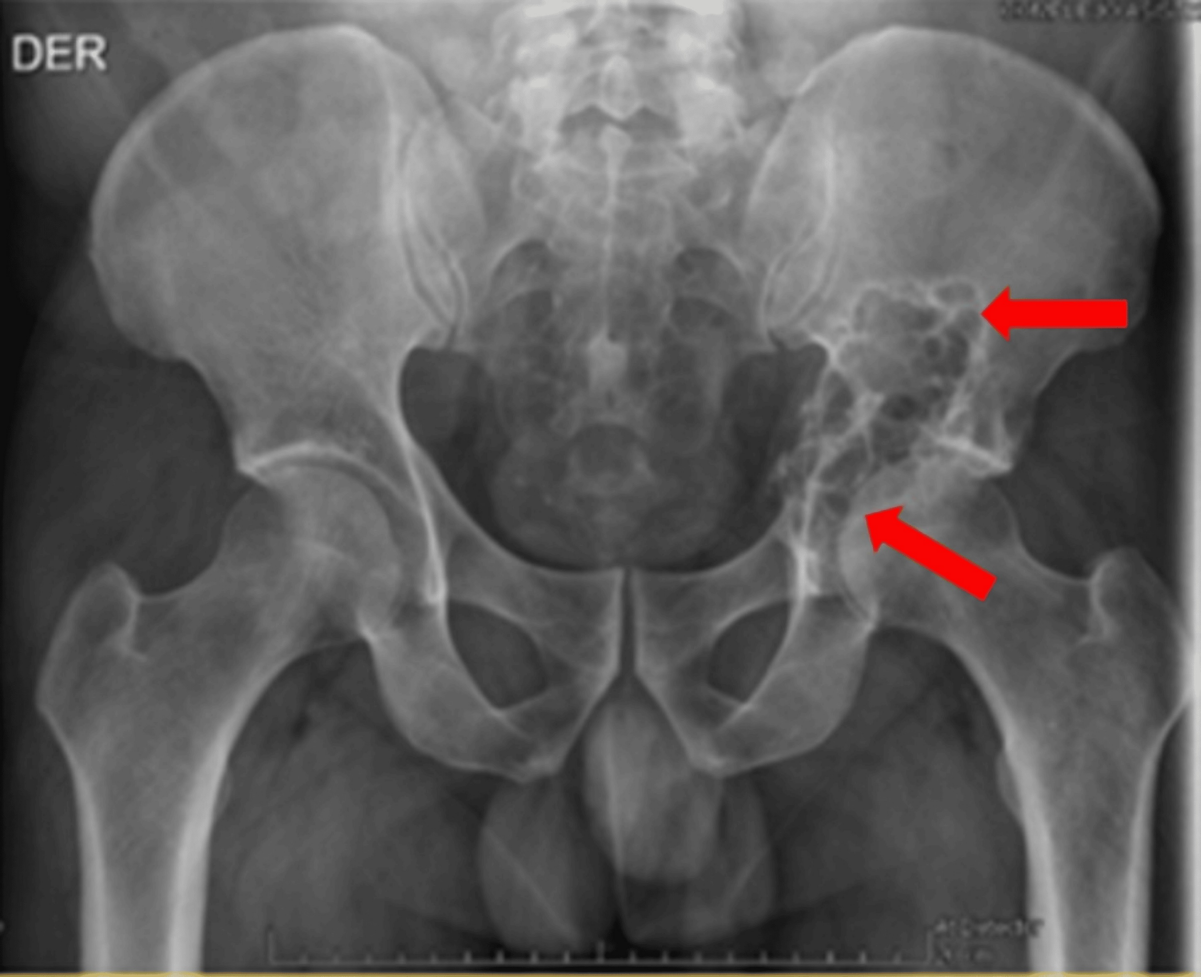
Initial pelvis X-ray. Anteroposterior pelvis radiograph showing an aggressive-looking lytic lesion (arrows) in the left acetabular region (zone III).

**Figure 2:**
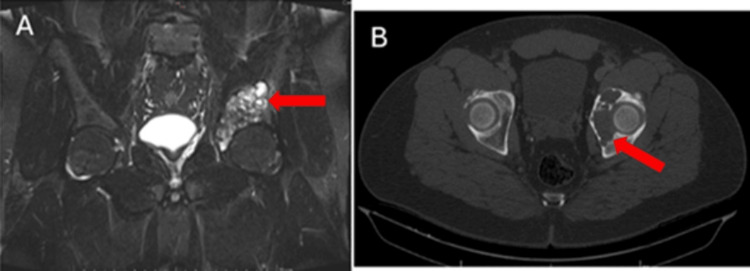
MRI and CT scan of the pelvis. (A) MRI of the pelvis, coronal section. (B) CT scan of the pelvis, transverse section, where an osteolytic lesion (arrows) is observed in the left hip region involving the acetabular roof.

A pelvic X-ray showed an osteolytic lesion involving the left acetabulum (Figure 1). Given the radiographic findings, the patient was hospitalized for further evaluation. Magnetic resonance imaging (MRI) of the pelvis and computed tomography (CT) of the thorax, abdomen, and pelvis were performed (Figures 2A, 2B). Imaging revealed a destructive, well-demarcated lesion centered in the acetabular dome, without periosteal reaction or matrix mineralization, consistent with a chondroid neoplasm. Differential diagnosis included low-grade chondrosarcoma, metastasis, or atypical degenerative changes. However, the absence of subchondral sclerosis and joint space narrowing made osteoarthritis unlikely.

**Figure 3:**
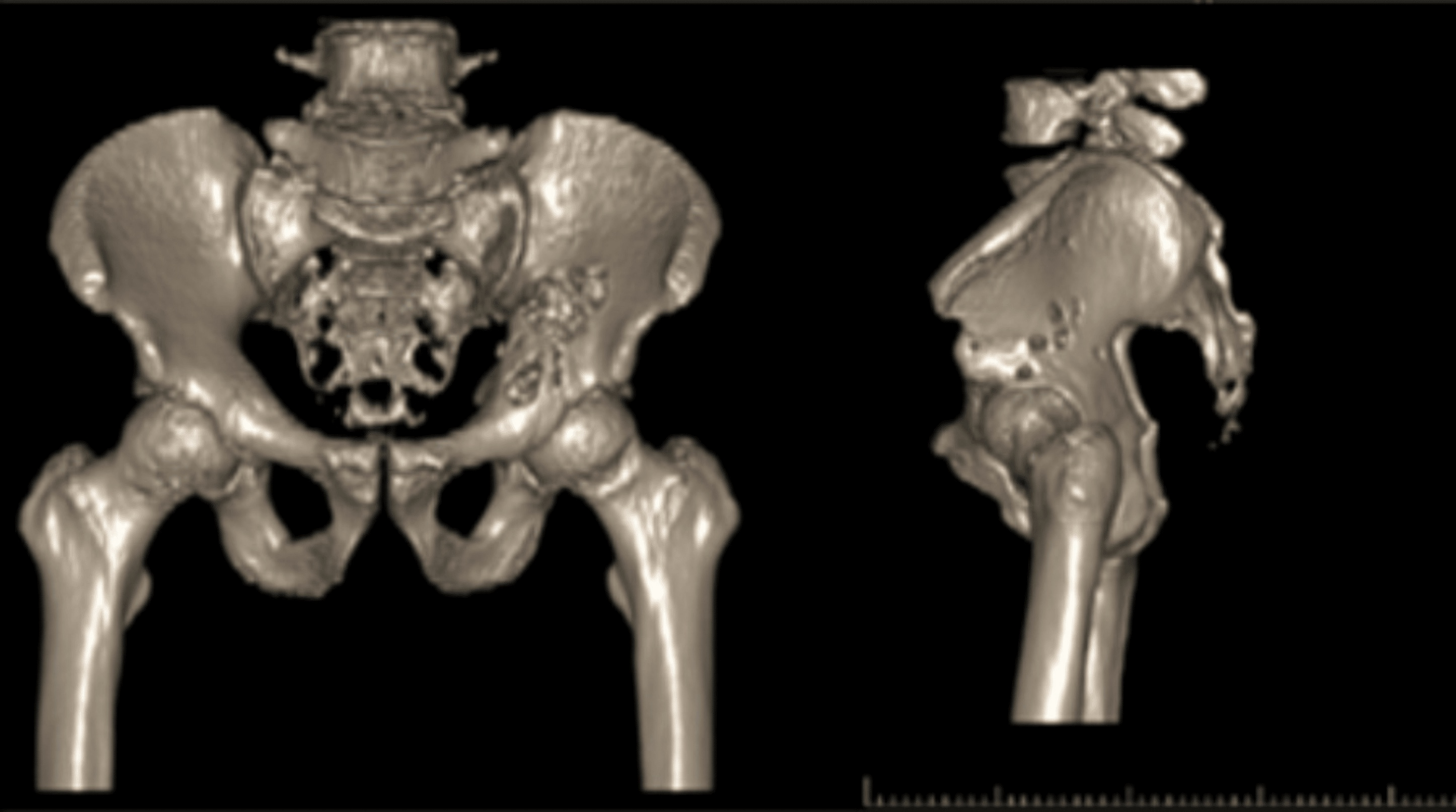
Pelvis CT scan with 3D reconstruction.

An open biopsy of the lesion confirmed a conventional low-grade chondrosarcoma (G1). A CT scan of the pelvis with 3D reconstruction was then obtained to produce a patient-specific printed model for surgical planning (Figure 3). Subsequently, the histological examination of the resected specimen revealed a grade 2 chondrosarcoma. This upgrade from the initial biopsy is clinically relevant, as higher-grade tumors are associated with a greater risk of local recurrence and metastatic potential. Although surgical planning was initially based on a low-grade diagnosis, the choice of a wide type II resection was appropriate and oncologically adequate for both scenarios.

The patient's post-operative course was favorable. At three weeks, he was pain-free and began physiotherapy. By three months, he initiated partial weight-bearing despite mild cephalic migration of the prosthesis. At six months, he remained stable, walking with a cane and without pain or neurological signs.

Surgical technique

**Figure 4:**
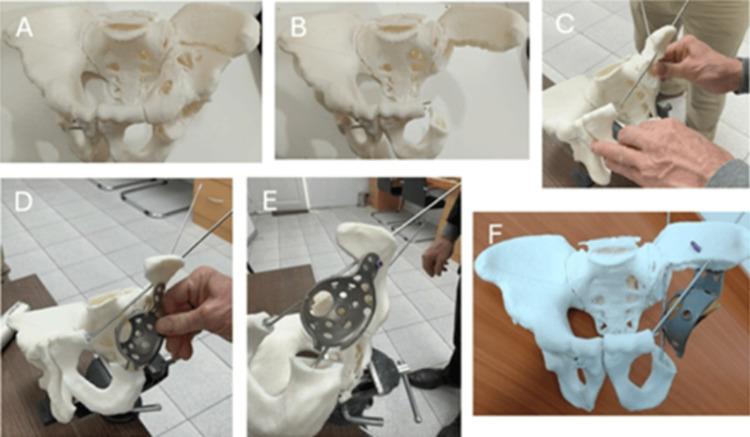
Step-by-step images of surgical technique. (A) 3D impression model of the patient's pelvis, in which the osteomies required for en bloc resection of the tumor lesion have been performed. (B) Step 1. (C) Step 2. (D) Step 4. (E) Step 5. (F) Step 6.

The patient was positioned in the right lateral decubitus position, and an iliofemoral approach was utilized to access both the endopelvic and exopelvic sides of the acetabular region. After performing an osteotomy of the femoral neck, the acetabular fundus was exposed, and the osteotomies pre-planned using a 3D model were executed, allowing for the en bloc resection of the acetabular segment. The chondrosarcoma was excised through precise osteotomies, including a proximal cut at the level of the iliac wing and additional cuts at the iliopubic and ischiopubic branches (step 1). Reconstruction commenced with the insertion of two hydroxyapatite-coated Schanz screws, oriented superior to inferior, which were placed from the iliac wing remnant to the iliopubic and ischiopubic branches to restore the continuity of the anterior and posterior columns (step 2). A basket was then cemented onto a frame formed by these Schanz screws (step 3). To enhance fixation, proximal screws were placed into the iliac wing remnant, and the femoral head was secured to the internal table using screws from the proximal fixation of the basket, increasing its length and providing better anchorage to the iliac wing (step 4). Finally, a dual mobility system was implemented (step 5), and a tapered femoral stem was inserted (step 6) to complete the surgical reconstruction (Figures 4A-4F).

The use of a dual mobility system was chosen to maximize hip stability and reduce the risk of dislocation, considering the altered biomechanics and limited bone support in this post-resection context. Moreover, this technique may be adapted to other types of pelvic tumor resections, as long as the anatomical conditions allow for stabilization with conventional implants planned through 3D modeling.

**Figure 5:**
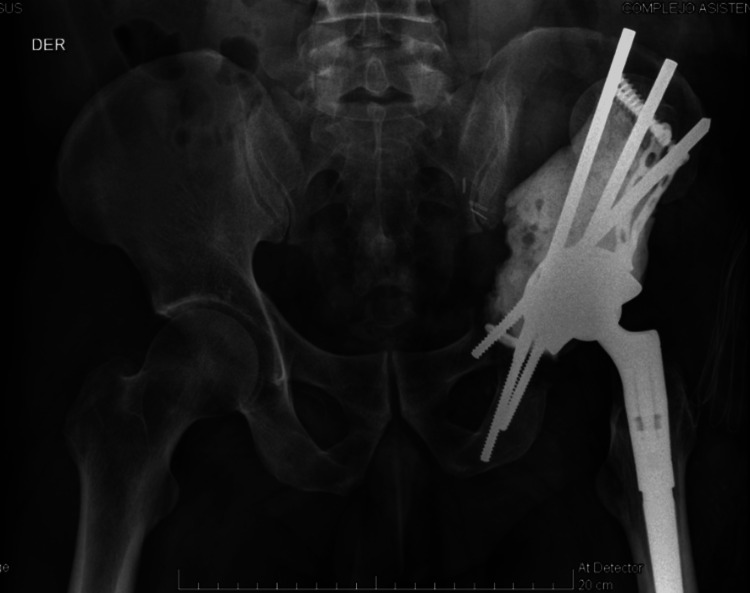
Immediate post-operative pelvis X-ray. Anteroposterior view showing the arrangement of the Schanz wire in anterior and posterior columns and the final reconstruction.

After this planning, November 17, 2023, was set as the date for surgery, which was successful. An immediate post-operative pelvis X-ray was performed (Figure 5). Regarding the new biopsy performed, the result shows morphological findings compatible with conventional chondrosarcoma, grade 2. During the four days following surgery, the patient evolves favorably without complications. He was discharged on the fourth post-operative day.

The first control in the traumatology polyclinic was performed after three weeks post-operatively. The patient was in good general condition, without pain, with adequate tolerance to walking, without complications. It was indicated to start kinesiotherapy without load.

**Figure 6:**
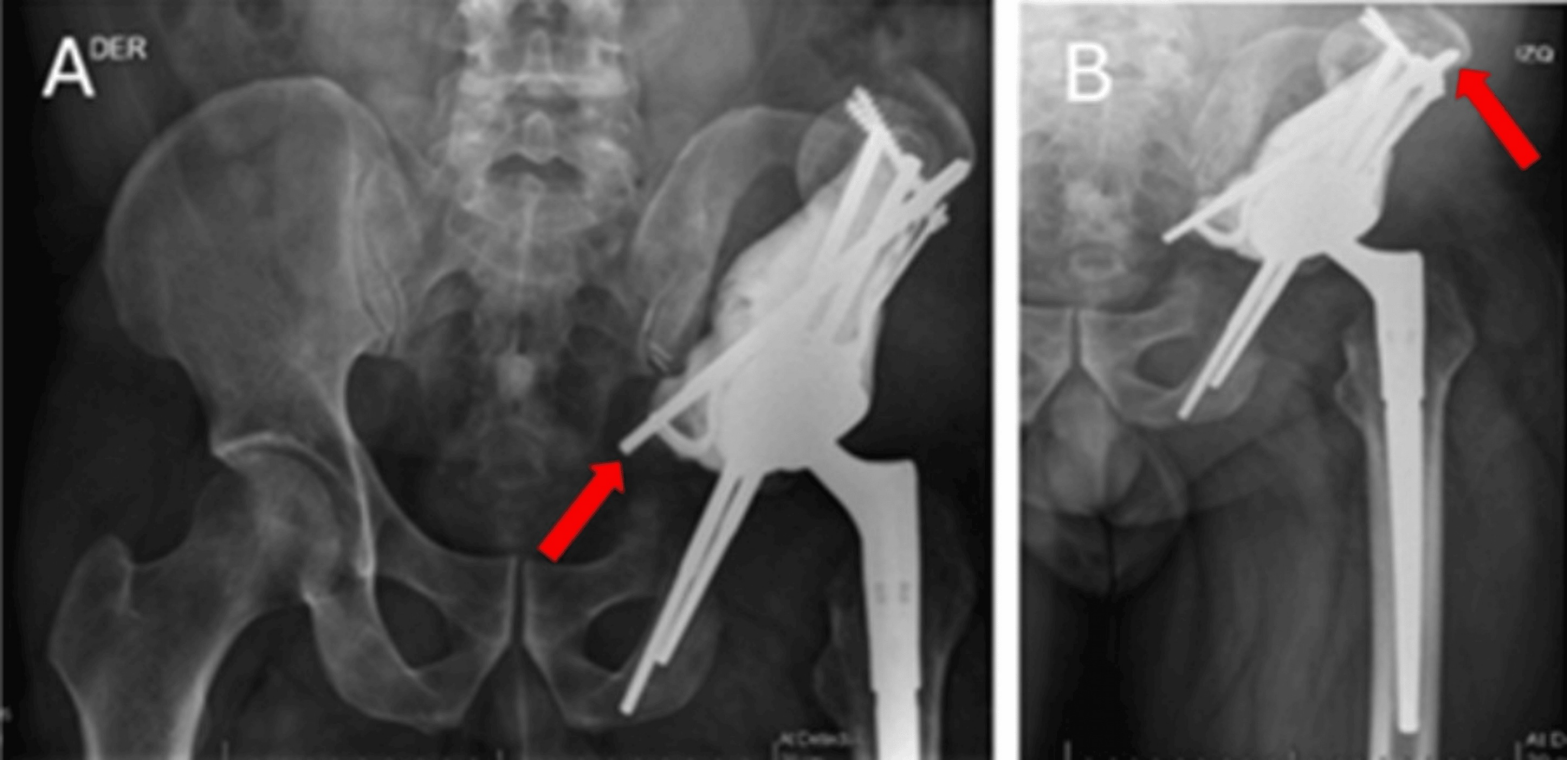
Post-operative X-ray at three months of control. (A) Anteroposterior pelvis X-ray. (B) Anteroposterior X-ray of the left hip. Remnant iliac wing fracture and slight elevation and medicalization of the hip are observed (Arrows). There is support for the construction towards the sacroiliac joint, achieving a stable situation.

Second control at three months post-operatively showed persistence with good clinical evolution and no pain. The control X-ray showed a slight cephalic displacement of the prosthesis (Figures 6A, 6B). It was recommended to progress in kinesiotherapy with a partial load of 10%, gradually increasing it according to the patient's tolerance.

At six months post-operatively, she was in good general condition, with a favorable clinical evolution, and was pain-free. She was able to ambulate using a cane and showed no signs of neurological compromise in the affected limb. The control X-ray revealed stabilization of the prosthesis without further cephalic migration.

The prosthesis displacement observed in this patient is an expected outcome, given the combination of factors such as loss of bone support, altered biomechanical loads, and shear forces acting on the prosthesis in a complex and compromised anatomical environment. This highlights the importance of regular radiological follow-up and a multidisciplinary approach to post-operative management, which optimizes functional outcomes and minimizes long-term complications.

## Discussion

Surgical treatment of pelvic chondrosarcoma, particularly those of acetabular location, is a significant challenge due to the tumor's resistance to conventional treatments such as chemotherapy and radiotherapy. Surgical resection remains the only viable therapeutic approach, but the anatomical complexity of the pelvis makes it difficult to achieve tumor-free surgical margins, with the consequent risk of local recurrence [11,12]. Moreover, reconstruction of the acetabular segment often involves the need to use unconventional or custom-made implants to restore anatomy and function. Even so, internal hemipelvectomy (limb-sparing) is generally preferred over external hemipelvectomy (or interilioabdominal amputation), if it is possible to preserve the limb and maintain a function that allows weight-bearing and ambulation [11].

Skeletal reconstruction techniques include the use of bone allografts and/or modular or unconventional prostheses. Both have high complication rates, including infections and implant failure [11,13]. Anatomic variability and the magnitude of bone loss increase the complexity of these procedures, so careful planning of the type of approach, the osteotomies required for resection with adequate margin, and the strategy for reconstruction are essential pre-operative considerations. The use of software tools for three-dimensional imaging has enabled more accurate planning of surgical techniques and also the construction of customized prostheses (CAD-CAM). Using 3D printing in a particular clinical situation, the surgeon can also "execute" the proposed surgery on a printed plastic model, selecting with less possibility of error and improvisation, both the surgical resection and the reconstructive method [11]. In the case presented, the design of the bony resection resulted in the preservation of pelvic ring structures that allowed reconstruction using Schanz wires and cement to restore the anterior and posterior acetabular columns, to which a conventional hip prosthesis was added. We believe that, despite fracture of the remaining iliac bone, the construct achieved a point of balance and support through discrete ascent and medicalization. Although the follow-up is brief, the patient continues to use only a cane without experiencing any pain. Although the technique was designed for an enneking type II resection, it could potentially be adapted to other pelvic resections involving zones I or III, provided that stable pelvic ring structures are preserved. The strategy of using conventional implants guided by 3D modeling offers a practical solution in settings without access to customized prostheses. In comparison to massive allografts or patient-specific endoprostheses, this technique may reduce costs and technical complexity, while still providing functional outcomes that are acceptable for daily mobility in selected cases.

On the other hand, there is the possibility of fabricating customized implants, based on the planned resection, and adapting the reconstruction exactly to the specific case. A recent study highlights that customized prostheses have shown superior results in terms of functionality and patient satisfaction compared to conventional techniques, especially in complex pelvic reconstructions [14].

Despite these advances, the implementation of advanced technologies in these situations is not without challenges, such as high costs and the need for specialized medical equipment in referral centers. In surgery, infections remain a major complication in pelvic reconstruction, due to their prolonged duration and the use of large implants [13]. The need for a multidisciplinary approach, including oncology, orthopedic surgery, and intensive postoperative care, is also crucial for improving outcomes and reducing complications. The latter emphasizes the importance of treating these patients at referral centers.

## Conclusions

The management of pelvic chondrosarcoma, especially in young patients, requires a multidisciplinary approach. The development of technologies for image processing and 3D model creation has improved the safety and precision of surgical planning. In the case presented, these tools enabled accurate resection and functional reconstruction using conventional implants. Although CAD-CAM systems allow for the design of customized prostheses, their cost and limited availability remain significant barriers. This report highlights a cost-effective and accessible alternative for managing acetabular resections in resource-limited settings. Notably, despite minor cephalic migration of the implant, the patient maintained good function, suggesting that with proper planning, conventional implants can achieve satisfactory outcomes. Future refinements might include enhanced anchoring strategies, integration of semi-custom components, or modular fixation systems to further improve implant stability. Long-term studies are needed to evaluate durability, optimize technique, and confirm reproducibility across centers.
